# Concussion injuries in sports and the role of instrumented mouthguards: a mini review

**DOI:** 10.3389/fbioe.2025.1567429

**Published:** 2025-04-01

**Authors:** Zaid Chilmeran, Muhammad Umair Akhtar, Abu-Baker Khalid Sharafeldin, Declan Gaynor

**Affiliations:** School of Medicine, Royal College of Surgeons in Ireland - Bahrain, Al Muharraq, Bahrain

**Keywords:** concussions, contact sports, instrumented mouthguards, sports medicine, head acceleration events

## Abstract

Contact sports such as American football, rugby, soccer, and ice hockey involve high-speed, high-impact interactions that frequently result in head acceleration events (HAEs), which can lead to concussions and other forms of traumatic brain injury. HAEs can lead to acute symptoms like dizziness and memory difficulties, as well as more severe, chronic conditions like cognitive decline and chronic traumatic encephalopathy. This mini-review focuses on concussion-related injuries in contact sports, examining their prevalence, impact, and the role of innovative prevention strategies. Particular attention is given to the development of instrumented mouthguards (iMGs), which incorporate real-time sensors to measure and analyze head impacts. Ultimately, this review aims to provide an overview of the role of iMGs on concussion prevention and its evolving landscape, with a focus on the potential of iMG technology.

## 1 Introduction

Many of the most popular sports today, such as American football, rugby, soccer, and ice hockey, are high-speed, high-impact contact sports that attract millions of participants. These sports often appeal to young athletes due to their physicality and excitement, but perhaps even more importantly due to various interpersonal factors such as social norms and team friendships (Howie et al., 2020). However, the very nature of these sports makes players susceptible to frequent head impacts, known as head acceleration events (HAEs), which can lead to concussions or traumatic brain injuries.

HAEs occur when the head undergoes sudden linear or rotational motion due to direct impacts to the head or through the transmission of forces from body contact, such as tackling or collisions (Kuo et al., 2022; Tierney, 2021). These events result in the brain moving rapidly within the skull, which can cause a concussion by stretching or damaging brain tissue and disrupting neural function. Studies have shown that in contact sports like American football, players can experience hundreds of sub-concussive HAEs over a single season (Bailes et al., 2013). Given that such repetitive incidents can lead to cognitive alterations (Walter et al., 2022), there is a valid concern over these repeated exposures.

The implications of concussions are serious, with long-term consequences that can include cognitive decline, memory deficits, depression, and in some cases, chronic traumatic encephalopathy (Martini and Broglio, 2018). Immediate effects of concussions may include loss of consciousness, dizziness, difficulty concentrating, and mild impairments in verbal memory (McCrea et al., 2003).

Given the serious risks associated with HAEs, preventing concussions and collecting data on how these injuries occur has become a priority for sports safety research. Traditionally, concussion prevention strategies have included the use of protective equipment like helmets and standard mouthguards, as well as rule changes, education and concussion anticipation aimed at reducing high-impact collisions (Schneider et al., 2017). In recent years, the development of instrumented mouthguards (iMGs) has introduced a new way to address concussion risks by embedding sensors within the mouthguard to monitor and quantify head impacts in real-time. Various sports, particularly contact sports like football, rugby, and even soccer—where heading the ball introduces potential for head trauma—stand to benefit from this technology.

**FIGURE 1:**
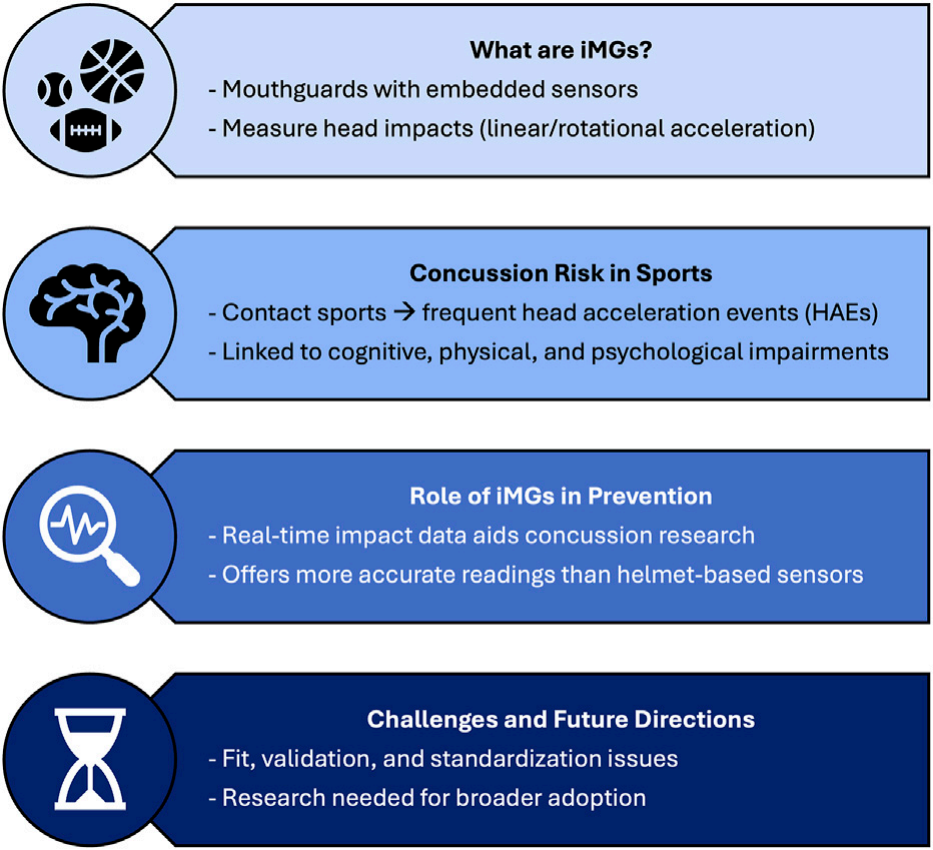
An overview of concussions and instrumented mouthguards in contact sports.

This mini-review aims to summarize various aspects of concussive injuries in sports, focusing on their prevalence, effects, and prevention strategies, particularly the role of iMGs in injury prevention (Figure 1). The review will first provide an overview of concussions, their causes, and current diagnostic methods. Next, it will discuss different preventative measures, with an emphasis on how mouthguards may help reduce concussion risk. Finally, the review will assess the effectiveness of these devices, including their validation and the challenges associated with measuring their impact.

## 2 Concussion injuries in sports

### 2.1 Pathophysiology and biomechanics of concussions

**FIGURE 2:**
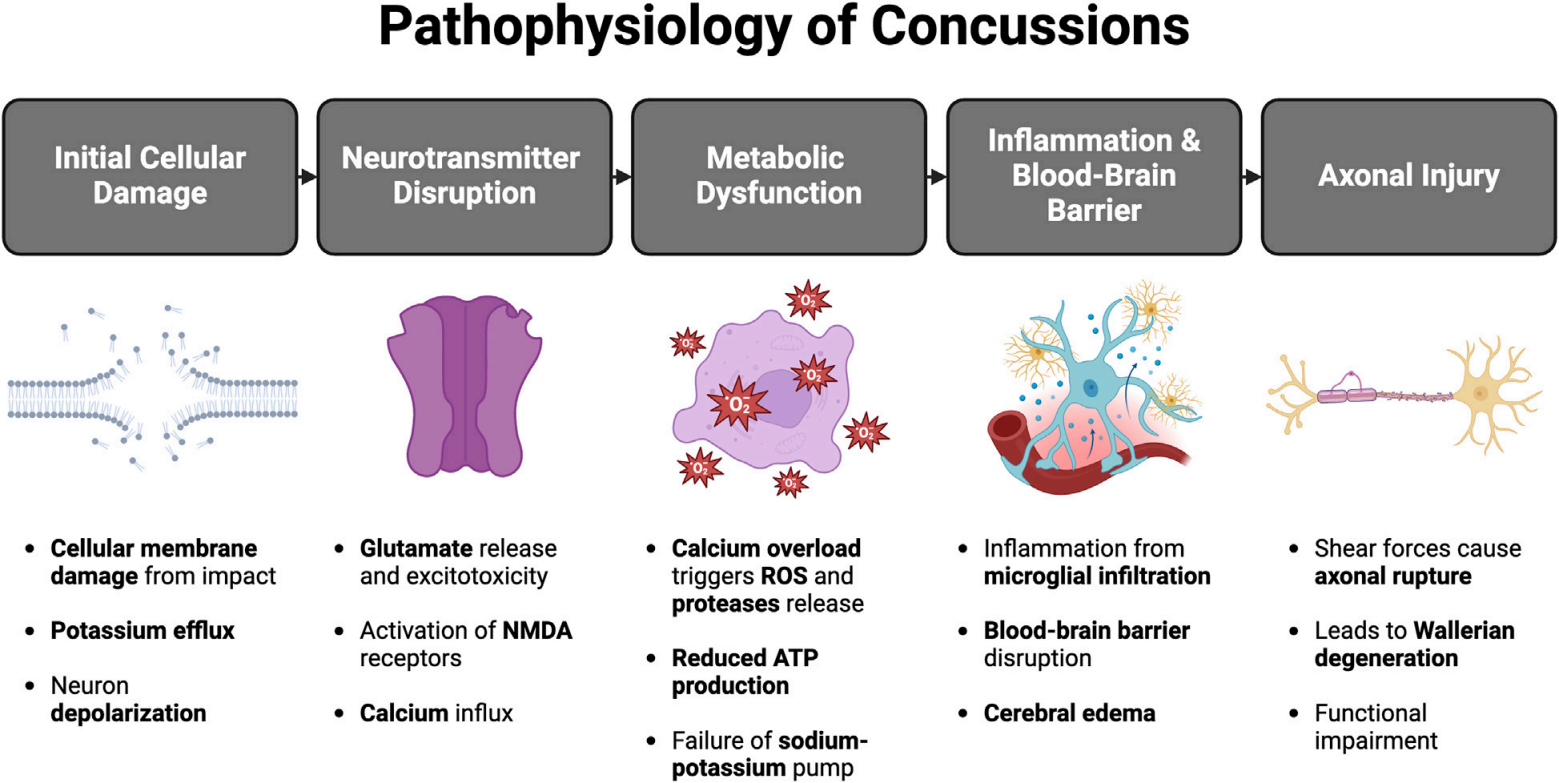
Events involved in the pathophysiology of concussion (Created in BioRender. C, Z. (2025) https://BioRender.com/n74g698)

The pathophysiology underlying the effects of concussions are mainly due to damages arising from abnormalities at the cellular level secondary to the impact (Figure 2). These include potassium efflux, calcium influx, blood-brain barrier damage, and axonal injury, all connected in a chronological order.

Initially, the trauma causes damage to the cellular membranes causing great efflux of intracellular potassium, leading to depolarization of the neuron. This causes a feedback loop secondary to the open voltage-gated channels. The feedback loop causes further release of intracellular potassium and depolarization of the neuron (Giza and Hovda, 2014). As a result, several neurotransmitters pour out of the damaged neuron cells, the main pathological neurotransmitter being glutamate. What makes this neurotransmitter most pathological is its ability to bind ligand-gated potassium ion channels which further lead to potassium efflux and depolarization (Katayama et al., 1990). Additionally, glutamate binds *N* -methyl- d -aspartate receptors to open Calcium-activated potassium channels, leading to the release of potassium and influx of calcium.

The accumulation of calcium in the cell causes additional cell cytotoxicity via activation of proteases, reactive oxygen species (ROS), and damage of the mitochondria (Cheng et al., 2012). Consequently, a metabolic mismatch ensues. The cells fail to produce ATP, leading to the failure of the ATP-dependent Sodium-potassium pump to maintain the correct ionic gradients (Hovda et al., 1990).

Glycolysis is activated due to the reduced ATP and increased energy demands which leads to lactic acid production; the accumulation of this product damages the blood-brain barrier causing massive cerebral edema (Verweij et al., 2007). Inflammation results from the abnormal metabolism led by microglial infiltration, although the response is local the release of cytokine mediators, proteases and ROS causes cerebral blood flow changes secondary to increased blood-brain barrier permeability (Steenerson and Starling, 2017; Loane and Byrnes, 2010).

Shear force secondary to direct blows to head or neck acceleration-deceleration injuries can lead to cytoskeletal injury which disrupts the accumulation and transport of products to the injured areas (Steenerson and Starling, 2017; Smith et al., 1999; Dixon et al., 1987). The disruption leads to further impaired metabolism and activation of cytokines with further aid in cytoskeletal damage.

In addition, axonal rupture from shear and tensile forces can result in Wallerian degeneration, transection, and even cell death, compromising cortical and subcortical pathways, and deleteriously reducing functional ability (Steenerson and Starling, 2017; Povlishock et al., 1992).

### 2.2 Effects of concussive events

Concussive events in high-speed contact sports can result in various symptoms and effects, both short-term and long-lasting. Most effects can be classified into three main types: psychological, cognitive, and physical.

Emotional and psychological changes are well-known sequelae of repeated concussions. Depression, a mental health disorder of low mood, was found to be of high incidence in those with a history of concussion. For instance, one study found a 3-fold increase in depression rates in those with previous concussions (Chrisman and Richardson, 2014). Similarly, a long-term National Football League (NFL) study found an increase in the proportion of physician-diagnosed depression in groups of higher concussion counts (Kerr et al., 2023). It was thought that a potential cause of depression arises because of the pathologic neuroinflammatory state of the brain post-trauma (Bodnar et al., 2018). Kohler et al. identified increased cytokine levels (IL-6 and TNF-a) in those with major depressive disorder compared to healthy individuals (Bodnar et al., 2018; Köhler et al., 2017) and demonstrated the usefulness of treatment through antidepressants to reverse this change (Köhler et al., 2018).

Cognition is another majorly affected component in those with a history of brain trauma. In terms of long-term cognitive impairment, more than half of children and adults displayed cognitive impairment after mild traumatic brain injury (mTBI), with prevalence slightly higher in adults (McInnes et al., 2017). Additionally, attention, memory, executive function, language, psychomotor function, intelligence, and perception were all reduced across studies when compared with control participants, normative data (Cunningham et al., 2020).

Physical effects, such as chronic headaches, are also amongst the common long-term manifestations of one with a history of concussion. A multicenter longitudinal survey of headaches and concussions among young athletes in the United States illustrated a strong association between a greater number of past concussions and a “more severe headache burden” (Ali et al., 2023).

### 2.3 Prevalence of head acceleration events in sports

The incidence of concussions is relatively common across a variety of sports. American football and hockey are the sports with the highest risk of concussion in men, soccer and basketball are among the highest risk of concussion for women (Ianof et al., 2014). American football has many players partaking in the sport, which contributes to the massive number of concussions in sport-related concussion epidemiology. For instance, an observational study conducted between 2015 and 2019 identified 1,302 concussions in 1004 NFL players, with the majority occurring during NFL games (Mack et al., 2021). Similarly, in the 2022 NFL season, 149 concussions were suffered across 271 games, giving a rate of around a concussion every two games (Seifert, 2017).

As for hockey, due to the high-speed the nature of the game, frequent physical contact, and the risk of players being struck by pucks or bodies, there is a high risk of injury. In fact, over 50% of National Hockey League (NHL) players report having to miss games due to significant injuries (Donaldson et al., 2014). Concussions are therefore a significant concern in hockey, making up 14%–30% of all head injuries in the sport (Andrews et al., 2022). In NHL, the rate of concussions is notably high, with approximately 5.8–6.1 concussions occurring per 100 games (Andrews et al., 2022). This prevalence is largely due to the high-speed nature of the game, frequent physical contact, and the risk of players being struck by pucks or bodies.

## 3 Mouthguards as concussion monitoring devices

### 3.1 History of mouthguards

The introduction of mouthguards in sports can be traced back to boxing, where athletes used makeshift devices made from cotton, tape, sponge or small pieces of wood (Knapik et al., 2007). These early designs, while primitive, highlighted the need for orofacial protection in sports. However, they were deemed illegal due to safety concerns as there were reported cases where materials were dislodged from the teeth and into the larynx (Knapik et al., 2007). The evolution of mouthguards can be attributed to dentist Woolf Krause, who in the late 19th century contributed with significant strides by developing a more functional mouthguard, later refined by his son Philip Krause (Knapik et al., 2007; Gould et al., 2019).

By the mid-20th century, other contact sports beyond boxing began recognizing the importance of mouthguards. In the 1962 season, the use of mouth protectors became mandatory for all high school students and some college football players, with many schools fabricating latex mouthpieces for their entire teams (Dukes, 1962). The National Alliance Football Rules Committee recommended that these models be custom-fit to the players, as they were the most effective and had the highest player acceptance rate. By this time, mouthguards could be fabricated inexpensively, quickly, and easily while maintaining their effectiveness (Association, 1962). Other sports, such as ice hockey, men’s lacrosse, and rugby, soon followed suit (Duffy, 2005; LaCrosse, 2005; Quarrie et al., 2005). Today, the American Dental Association recommends mouthguards for use in over 29 sports and exercises, underscoring their critical role in athlete safety (Association, 2004; Policy on Prevention of Sports-related Orofacial Injuries, 2018).

Over time, mouthguards have evolved significantly in their design and material composition, offering better protection for athletes against orofacial trauma. The type of material has changed, such as ethylene vinyl acetate (EVA) being substituted for rubber (Patrick et al., 2005), becoming the most popular material used (Mascarenhas, 2012). Most mouthguards are now constructed out of EVA, which paved the way for custom-fit mouthguards, where a meta-analyses of 12 cohort trials and 11 self-report questionnaires from a variety of sports showed that the overall risk of an orofacial injury was more than twice as great when athletes were not wearing a mouthguard (Knapik et al., 2019).

The protective capability of mouthguards and mouthguard materials are generally indicated by shock absorbing capability, hardness and stiffness. Mouthguard durability is generally indicated by tensile strength and tear strength, while mouthguard stability is generally indicated by water absorption (Knapik et al., 2007). However, the current evidence indicates that mouthguards have little impact on preventing concussions (Mascarenhas, 2012; Knapik et al., 2019), with inconsistent and unclear data (Mascarenhas, 2012). This is because while mouthguards reduce head acceleration, this alone does not directly correlate with a reduced risk of concussions (Mascarenhas, 2012).

### 3.2 Development of instrumented mouthguards

In recent years, several advanced devices have emerged to monitor head impacts in sports, aiming to enhance concussion detection and prevention. Among the most commonly used systems are the Head Impact Telemetry System (HITS), which is embedded in helmets, and the X2 Biosystems X-Patch, a sensor worn behind the ear (O'Connor et al., 2017). A newer innovation, iMGs, utilizes accelerometers to measure acceleration-deceleration and peak linear acceleration (PLA), and gyroscopes to assess rotational forces and peak rotational acceleration (PRA) (King et al., 2015). iMGs stand out when compared to HITS and the X2 Biosystems X-Patch due to their strategic placement. Helmet-mounted sensors like HITS can misrepresent forces with overestimations and underestimations, high error rates, and low specificity in predicting concussive injury (Beckwith et al., 2012; Jadischke et al., 2013; O'Connor et al., 2017). While skin-mounted sensors, such as the X-Patch, introduce many inaccuracies and should be used only for research purposes, ideally in combination with video analysis (McIntosh et al., 2019). In contrast, iMGs deliver more accurate and reliable measurements of both linear and rotational accelerations (Stitt et al., 2021; Gabler et al., 2021), offering a more precise understanding of head impact biomechanics. However, these studies tend to assume the head to be a rigid body, which may not always reflect the true dynamics of head motion during actual sports impacts (Stitt et al., 2021).

Traditionally, regulatory safety standards used 3 degrees of freedom (3DOF) translation-only kinematic criteria (Hernandez et al., 2015). iMGs that measure six degree of freedom (6DOF) contain a tri-axis accelerometer, as well as a tri-axis gyroscope (Gellner R. et al., 2024), and are necessary to investigate human mTBIs due to them being better predictors of injury compared to 3DOF translation-only and rotation-only criteria (Hernandez et al., 2015).

### 3.3 Validation

To ensure reliable data collection across various sports and impact scenarios, it is crucial to validate the accuracy of iMGs. Studies have compared iMG readings with those from traditional helmet-mounted sensors, like the HITS, to assess their effectiveness in measuring head impacts. In one study, 133 paired events were recorded during a high school football season, comparing the HIT System with an iMG. While the average errors between the two systems were low, there was significant variability, particularly during higher-impact events (Holcomb et al., 2024).

Another study in varsity football examined 53 head impacts and found that while average brain strain remained consistent across different time windows, some impacts showed up to 40% variability. This was especially true for impacts with lower peak accelerations (Bouvette et al., 2024). The study emphasized the importance of standardizing sampling times, as another study shows that longer time windows in mouthguards led to more reliable brain strain calculations, with errors averaging under 9% (Liu et al., 2020).

Further validation efforts compared iMGs with reference sensors on helmeted headforms, revealing strong correlations with minimal error. One study found mean absolute errors as low as 7% (Gabler et al., 2021). In addition, another study showed that iMGs performed consistently well in measuring linear acceleration, angular velocity, and angular acceleration, with minimal variability (Rich et al., 2019). However, iMGs tend to lack validity under high-velocity impacts, which highlights the importance of cadaveric testing to accurately replicate real head kinematics, jaw dynamics, mandible fit, and secondary impacts (Abrams et al., 2024). In real-world settings, such as with 915 female youth soccer players, iMGs showed sensitivity of 69.2% and a positive predictive value of 80.3%, confirming their reliability for on-field use (Rich et al., 2019). Additionally, implementation of machine learning and algorithms into iMGs can significantly increase precision (Gabler et al., 2020), and also accuracy when identifying impact locations (Sohail et al., 2022).

The accuracy of iMGs can be influenced by both filter cutoff frequencies and proper coupling with the athlete’s skull. Signal filters often operate below recommended standards to manage noise in live data, with research indicating that optimal cutoff frequencies of 175 Hz for linear acceleration and 250 Hz for angular velocity reduce measurement errors (Gellner R. et al., 2024). Additionally, poor coupling between the iMG and the skull can result in significantly higher peak angular acceleration and increased signal noise, even in confirmed impact events. A study with university athletes suggested using infrared proximity sensing to identify poorly coupled events, which, when combined with video analysis, could help minimize sensor noise and improve data quality (Luke et al., 2024).

Fit also plays a crucial role in iMG performance. A study on decoupling (gaps between the mouthguard and the jaw) found that larger gaps increased measurement errors, particularly during frontal impacts. This highlights the importance of proper fit for improving data accuracy (Gellner RA. et al., 2024).

A recent study suggested that deviations of tested iMGs from reference values may result from differences in sensor placement and mouthguard design, highlighting the need to validate iMG usage *in vivo*. This is particularly important when considering realistic (non-idealized) sensor-skull coupling, interactions with the mandible, and subject-specific anatomy that may affect device performance (Abrams et al., 2024). Furthermore, varying sensor calibration methods across studies contribute to differences in results. For instance, Bartsch et al. applied post-process corrections to mitigate signal attenuation from over-filtering and thus achieved closer matches of angular acceleration (Bartsch et al., 2014), whereas another study that did not apply substantial calibrations resulted in less accurate values (Kuo et al., 2016). This inconsistency underscores the need for standardized calibration techniques to ensure reliable data interpretation and comparability across studies.

## 4 Discussion

### 4.1 Controversies

Despite their potential, the use of iMGs in sports biomechanics remains a topic of ongoing debate. Female and younger athletes are under-represented in the research to date, and the lack of standardized, independent validation protocols for these technologies is a significant concern (Tierney, 2021; Le Flao et al., 2022). Without such protocols, some suggest that substandard data could infiltrate the scientific literature, potentially misinforming clinical practices and undermining confidence among stakeholders, including healthcare professionals (Tierney, 2021).

Further complicating the issue is the current stage of technological development. According to a recent consensus statement, iMGs are still considered research tools that require additional refinement and validation before they can reliably be applied in clinical or field settings (Rivara et al., 2020). Additionally, physical factors challenge the accuracy of IMGs. Factors such as improper fit, direct contact, and interference within the mouth—caused by actions like biting, shouting, or contact with the lower dentition—can significantly compromise the reliability of the data (Tierney, 2021). These variables are difficult, if not impossible, to replicate using standard test dummy headforms, further complicating the validation process.

### 4.2 Clinical implications

iMGs are designed to measure head impacts by detecting high-magnitude accelerations that may indicate a risk of concussion. An injury metric known as the combined probability of concussion calculates the likelihood of a concussion occurring based on peak linear and rotational accelerations experienced during impact (Rowson and Duma, 2013). Similarly, sensor acceleration events, which occur in response to iMG sensors detecting head acceleration, can alert clinicians that there is an increased risk of concussion (Tooby et al., 2024). Such metrics can be used to aid clinicians in diagnosis of concussive events and thus provide care in a timely way. However, since the clinical response to a HAE is confounding by various other factors such as previous concussion history and head size, data from iMGs alone cannot be used to diagnose concussions (Tooby et al., 2024). A clinical diagnosis is made through the judgement of a clinician, often using assessment tools such as the Sport Concussion Assessment Tool sixth Edition (SCAT-6) (Echemendia et al., 2023a).

Concussions are diffuse injuries that usually do not present with focal neurological deficits, such as pupillary changes or signs of specific limb impairment. Rather, they often cause non-specific symptoms like dizziness, which makes early diagnosis challenging and requires the expertise of a specialist (Tator, 2013). Diagnostic approaches for concussions include imaging techniques such as MRI, fMRI, and CT scans. However, these methods may appear normal even after a concussion. Other diagnostic tools include assessment systems like the Balance Error Scoring System (BESS) and the SCAT, which are considered the current gold standard (Tator, 2013). It is important to note that these scoring systems should not be used as standalone diagnostic methods. Expert judgment is essential, as athletes may achieve a normal SCAT-6 score but still suffer from a concussion (Echemendia et al., 2023b).

Headgear sensors with gyroscopes and accelerometers, including iMGs, can be used to measure head movement and rotation during sports activities and predict the concussion risk of individual athletes (O'Connor et al., 2017). Newer devices offer several advantages, including real-time data, reduced underreporting of symptoms (as athletes may avoid reporting symptoms to continue playing), and the ability to predict and prevent concussions earlier. They also provide accurate, objective data, which traditional diagnostic methods, like scoring systems, often lack. However, a systematic review of head-impact measurement devices found that while these devices deliver real-time data and accurate head-movement kinematics, they have limited clinical utility. This is due to high error rates, low specificity in predicting concussions, and insufficient sensitivity compared to traditional scoring systems like SCAT-6 (O'Connor et al., 2017).

### 4.3 Feasibility and practical considerations

Tierney et al. outlined a four-phase protocol to assess the validity and feasibility of iMGs for quantifying HAEs, emphasizing the need for independent scientific validation due to the increasing emergence of iMG technology (Tierney et al., 2021). Phases three and four focus on evaluating feasibility from the perspectives of players and practitioners, respectively, to support the adoption of iMG systems (Tierney et al., 2021). Building on this protocol, one study applied it to four iMGs from different manufacturers and found that two of them received similarly high ratings for fit, comfort, and function (Jones et al., 2022). This study provided the first comprehensive analysis of iMG strengths and limitations, and offered valuable insights for sporting organizations to optimize hardware, software, and adoption practices. Additionally, a recent survey identified key barriers to iMG adoption, including discomfort, poor fit, and players’ preference for personal or no mouthguards, particularly during training. Inconsistent usage and concerns about performance impact also hinder adoption, suggesting the need for education and potential mandates from governing bodies (Roe et al., 2024). These studies highlight that comfort, proper fit, and athletes’ personal preferences are crucial factors for stakeholders to consider, as they will determine whether teams and athletes adopt this technology.

## 5 Conclusion

Concussions remain a significant concern in contact sports, where head impacts are frequent and often unavoidable. The long-term consequences of these injuries, including cognitive impairments, psychological disorders, and physical ailments, underscore the importance of prevention and effective monitoring. While traditional safety measures such as helmets and mouthguards have played key roles in injury mitigation, the introduction of iMGs offers advancement in concussion prevention and impact monitoring. These devices provide valuable real-time data on head accelerations, which can enhance our understanding of concussion mechanics and improve injury detection. However, despite the potential of iMGs, their widespread adoption is hindered by challenges in validation, consistency, and standardization. Studies have shown that while iMGs can accurately capture head impact data, their performance can be affected by factors such as fit, movement within the mouth, and environmental conditions. As such, more research is needed to refine these devices, develop standardized protocols, and ensure their reliability in both laboratory and real-world settings.

## Abbreviations

HAE, Head Acceleration Event; iMG, Instrumented Mouthguard; ROS, Reactive Oxygen Speices; ATP, Adenosine triphosphate; NFL, National Football League; US, United States; IL, Interleukin; TNF, Tissue Necrosis Factor; mTBI, Mild Traumatic Brain Injury; NHL, National Hockey League; ADA, American Dental Assosciation; EVS, Ethylene Vinyl Acetate; HITS, Head Impact Telemetry System; PLA, Peak Linear Acceleration; PRA, Peak Rotational Acceleration; DOF, Degrees of Freedom; SCAT6, Sport Concussion Assessment Tool 6th Edition.
